# Single cell transcriptomics reveals recent CD8T cell receptor signaling in patients with coronary artery disease

**DOI:** 10.3389/fimmu.2023.1239148

**Published:** 2023-09-27

**Authors:** Shahad Iqneibi, Ryosuke Saigusa, Amir Khan, Mohammad Oliaeimotlagh, Sujit Silas Armstrong Suthahar, Sunil Kumar, Ahmad Alimadadi, Christopher P. Durant, Yanal Ghosheh, Coleen A. McNamara, Catherine C. Hedrick, Klaus Ley

**Affiliations:** ^1^ Immunology Center of Georgia, Augusta University, Augusta, GA, United States; ^2^ La Jolla Institute for Immunology, La Jolla, CA, United States; ^3^ Cardiovascular Research Center, Cardiovascular Division, Department of Medicine, University of Virginia, Charlottesville, VA, United States; ^4^ Department of Physiology, Augusta University, Augusta, GA, United States

**Keywords:** atherosclerosis, CAD, tolerance, CD8 T cells, scRNA-seq, CITE-Seq

## Abstract

Coronary artery disease (CAD) is a major cause of death worldwide. The role of CD8+ T cells in CAD is unknown. Recent studies suggest a breakdown of tolerance in atherosclerosis, resulting in active T cell receptor (TCR) engagement with self-antigens. We hypothesized that TCR engagement would leave characteristic gene expression signatures. In a single cell RNA-sequencing analysis of CD8+ T cells from 30 patients with CAD and 30 controls we found significant enrichment of TCR signaling pathways in CAD+ subjects, suggesting recent TCR engagement. We also found significant enrichment of cytotoxic and exhaustion pathways in CAD cases compared to controls. Highly significant upregulation of TCR signaling in CAD indicates that CD8 T cells reactive to atherosclerosis antigens are prominent in the blood of CAD cases compared to controls.

## Introduction

Atherosclerosis is caused by hyperlipidemia that induces a chronic inflammatory state in the neointima of large and medium-sized arteries, which can lead to plaque buildup. Coronary Artery Disease (CAD) ensues when atherosclerosis affects the coronary arteries and restricts the blood supply to the heart, becoming a major cause of morbidity and mortality (1). When a coronary artery plaque ruptures or erodes, a myocardial infarction and acute death may result (2).

CD8+ T cells are found in large number in atherosclerotic plaques in humans and mice and have been proposed to be dysfunctional immune regulators of the disease (3). In mouse models of atherosclerosis, atherosclerotic lesions are found in the aortic sinus, the lesser curvature of the aortic arch, and at bifurcations (4). Different subsets of CD8 T cells with potential atheroprotective (5) and proatherogenic roles (3) have been described in the literature. Fernandez et al. (6) studied immune cells of patients with symptomatic or asymptomatic carotid artery disease using mass cytometry (CyTOF) and scRNA-seq and reported that CD8 T cells were abundant and more activated in carotid plaques compared to blood from the same subjects. In another study, atherosclerosis was associated with CD8 T cells killing other cells through granzyme B-mediated mechanisms, which was proposed to result in subsequent inflammation via TNF secretion (7). Meanwhile, CD95+CD8+ stem cell memory T cells were associated with more advanced CAD in a flow cytometry study (8).

Antigenic peptide epitopes for CD8+ T cells are restricted by Major Histocompatibilty Complex (MHC-I). Naïve CD8 T cells become effector cytotoxic T lymphocytes (CTLs) by cross-presentation on antigen-presenting cells (9). CTLs can kill cells that present cognate antigenic peptides on MHC-I, which is expressed on all nucleated cells. The antigen specificity of CD8 T cells in atherosclerosis is unknown. Chowdhury et al. (10) reported that human atherosclerotic plaques contained antigen-experienced memory CD8+ T cells. TCR sequencing revealed that some of these cells had TCRs specific for viruses such as influenza and coronavirus, suggesting potential cross-reactivity between viral and self-epitopes. Another recent study found that CD8 T cells in human carotid endarterectomy specimens were enriched in CD69, a marker of recent activation, or tissue resident memory (TRM) compared to CD8 T cells in PBMCs from the same patients. Transcriptomic analysis revealed expression of FOS and FOSB, also suggesting recent TCR engagement (11).

Atherosclerosis is associated with a break of tolerance to self-antigens. Wolf et al. (12) showed that the autoimmune response in patients and mice with atherosclerosis turned pro-inflammatory and exacerbated the disease process. Saigusa et al. (13) used scRNA-Seq with TCR-Seq and found that CD4 T cells specific for an apolipoprotein B epitope had transcriptomes similar to memory T cells in subjects with subclinical carotid artery disease. Depuydt et al. (11) performed single cell T Cell Receptor (TCR) sequencing on 3 patients with carotid artery disease and demonstrated clonal expansion of CD4 and CD8 T cells in plaque. In the most recent study, Wang et al. (14) showed clonal expansion of CD4+, CD8+, and regulatory T cells in the plaques and arterial tertiary lymphoid organs (ATLOs) of mice with atherosclerosis, accompanied by aberrant tolerance-regulating transcripts and dysfunctional antigen presentation. In the same study, scRNA-seq of human plaques also revealed CD8+ T cell tolerance dysfunction. Taken together, these studies support the notion that atherosclerosis is associated with an autoimmune response (15) caused by broken tolerance to self-antigens.

Based on these studies, we hypothesized that the blood of patients with CAD may contain CD8 T cells that were recently exposed to self-antigens. Such T cells would be expected to show signs of increased TCR signaling. Here, we investigated the peripheral blood mononuclear cell (PBMC) transcriptomes and surface phenotypes of 60 males and females with or without CAD and analyzed their transcriptomic changes with a focus on TCR signaling.

## Results

### Patient and cell populations

The 60 CAVA participants (16) selected for the study included subjects ranging from 42 to 78 years old, and mostly of non-Hispanic White/Caucasian descent. Categorical variables were tested by Chi-square test and continuous variables by Mann-Whitney test. **Table S1A** shows the 60 participants matched based on CAD status. Patients with Gensini scores exceeding 30 were categorized as having high CAD severity, those with a Gensini score below 6 were classified as low CAD severity subjects (controls). Participants were controlled for variables including smoking, statin use, and diabetes status. The majority of subjects were statin-treated. Lab results and current medications were considered. **Table S1B** shows the demographics, medications and labs comparing male and female participants. Significantly more women were on calcium channel blockers, and women had significantly higher high sensitivity C-Reactive Protein (hsCRP) values. The peripheral blood mononuclear cells (PBMC) tubes were transported on liquid nitrogen from the central repository; once thawed, they were processed using standard procedures. Excellent cell viability was achieved (90 ± 4%) (**Table S2**). To avoid batch effects, all cells were hash-tagged for multiplexing, with 4 samples run per 250,000-well plate (total of 16 plates). The pooled cells were labeled with 49 oligonucleotide-tagged titrated monoclonal antibodies (**Table S3**). After undergoing rigorous quality control steps, including a three-stage doublet removal, a total of 16,532 CD8+ T cells were identified, re-clustered, and analyzed.

### Differentially expressed genes by CAD disease status

**Figure 1:**
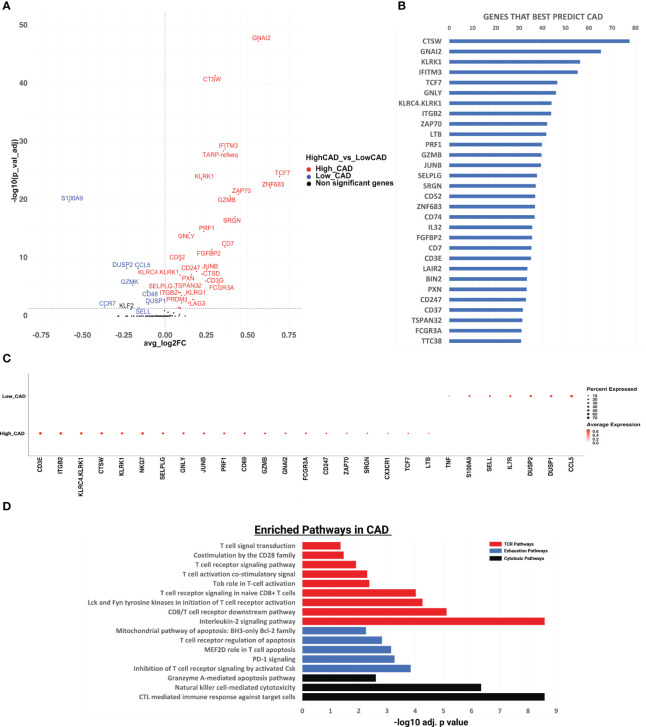
Gene expression analysis and pathway enrichment analysis of CD8+ T cells in cases vs controls. **(A)** Volcano plot showing the gene expression in all CD8+ T cells in CAD high vs CAD low. We performed differential expression analysis using Seurat’s FindMarkers function to extract marker genes. Significant markers were selected based on Bonferroni-adjusted P values <0.05. Colored dots (upregulated genes in red, and downregulated genes in blue) indicate significantly differentially expressed genes. Horizontal dashed line indicates a cutoff of an adjusted P value of 0.05. **(B)** The random forest feature importance plot showing the important genes contributing to CAD. The top 50 genes and their importance score are shown on the x-axis. The resultant “importance scores” were scaled to the 0-100 range for a better comparison. A higher score means that the gene is more important for classifying the groups (CAD cases vs controls). **(C)** Dotplot showing gene expression changes among CAD high vs CAD low. The size of the dot encodes the percentage of cells expressing each gene within each phenotype (CAD high or CAD low), while the color encodes the AverageExpression level across all cells within each phenotype. **(D)** Pathway enrichment analysis of the genes significantly upregulated in CAD using the Enrichr pathway analysis suite. x-axis shows the significance level based on Bonferroni - log10 adjusted p value. T-cell receptor (TCR), exhaustion, and cytotoxic pathways shown respectively in red, blue, and black.

We identified CD8 T cells as CD19-CD3+CD8+CD4-, reclustered them and analyzed gene expression differences in subjects with CAD vs. matched controls (**Figure 1A**). In all CD8+ T cells, 35 of 485 expressed genes tested, including granzyme B (GZMB), perforin 1 (PRF1), and ZAP70, a tyrosine kinase that gets activated and released upon T cell receptor engagement (17), were significantly upregulated in CAD+ patients. TCF7, a transcription factor involved in T cell development and memory, had the highest log-fold change in CAD cases vs. controls. TCF7 was previously found to be highly expressed in both mouse and human subjects with atherosclerosis, induced via the proinflammatory NFκB/AKT pathway (18). GNAI2 (encoding the G Protein subunit α_i2_) and CTSW (Cathepsin W) were the most highly significantly (p< 10^-40^) differentially expressed genes. The Gαi2 subunit is involved in chemokine receptor signaling (19). CTSW is a protease expressed in effector CD8+ T cells that is released during cell killing (20).

### Random forest

Next, to identify the genes whose expression can best distinguish between CAD+ and CAD-groups, we used a Random Forest machine learning (ML) approach. CTSW, GNAI2, KLRK1, IFITM3, and TCF7 were the most important genes in separating CAD from non-CAD participants (**Figure 1B**). Among the top 10 genes were 5 genes encoding cytotoxic proteins: CTSW, KLRK1, GNLY, KLRC4 and KLRK1 and two T cell receptor (TCR) signaling genes, ZAP70 and ITGB2, which encodes the beta chain of LFA-1, a key component of the immunological synapse (21). For the top genes identified by the random forest algorithm, we constructed a dot plot of expression (**Figure 1C**). The TCR signaling genes CD3E, ITGB2, JUNB and ZAP70 were highly expressed in many cells from CAD+ patients. KLRC4, KLRK1, CTSW, GNLY, PRF1 and GZMB, all encoding cytotoxic proteins, were also significantly overexpressed in CD8+ T cells from CAD+ patients.

### Pathway analysis

In subjects with CAD, we found TCR signaling, cytotoxic, and exhaustion pathways to be significantly upregulated. Nine pathways related to CD8 TCR signaling pathway were significantly enriched (**Figure 1D**), including the interleukin-2 signaling pathway, the CD8 TCR downstream pathway, Lck and Fyn tyrosine kinases in initiation of T cell receptor activation, T cell receptor signaling in naïve CD8+ T cells, Tob role on T-cell activation, T cell activation co-stimulatory signal, T cell receptor signaling pathway, costimulation by the CD28 family, and T cell signal transduction. This is a key finding, because recent work has provided evidence that T cells are repeatedly exposed to self-antigens in subjects with atherosclerosis (6, 10, 12–14). The highly significant enrichment suggests that in CAD+, but not CAD- subjects, some CD8 T cells have recently been exposed to antigen and activated their downstream TCR signaling pathway. To assess directionality, these genes were analyzed using IPA (Ingenuity Pathway Analysis), as shown in **Figure S1**. Again, the TCR signaling pathway was the most significantly positively enriched pathway, and the NFAT pathway (downstream of TCR) was second. All genes defining each significantly enriched pathway are shown in **Table S4 + Table S5**, with the significantly upregulated genes shown in bold.

Three cytotoxic pathways were also significantly enriched in CAD cases vs. CAD- controls (**Figure 1D**): CTL-mediated immune response against target cells, natural killer cell-mediated cytotoxicity and granzyme A-mediated apoptosis. The cytotoxic T lymphocyte pathway was the third-most significantly enriched pathway in IPA (**Figure S1**). This observation is consistent with (6) who reported CD8 T cell enrichment in the plaque and suggested that CD8 T cells may kill target cells in human carotid plaque.

Repeated or continuous TCR engagement may lead to CD8+ T cell exhaustion, a phenomenon well known to be involved in failure of cancer immunotherapy and in autoimmune diseases (22). Gene signatures from carotid plaque CD8 T cells showed evidence of exhaustion (6), although this finding was not confirmed in a second study (23). Here, we found significant enrichment of 5 exhaustion-related pathways, including inhibition of T cell receptor signaling, PD-1 signaling, MEF2D role in T cell apoptosis, T cell receptor regulation of apoptosis, and the mitochondrial pathway of apoptosis (**Figure 1D**). The genes contributing to this pathway included PRDM1 and LAG3, which are overexpressed in exhausted T cells (24) (**Table S5**).

### CD8 T cell clustering

**Figure 2:**
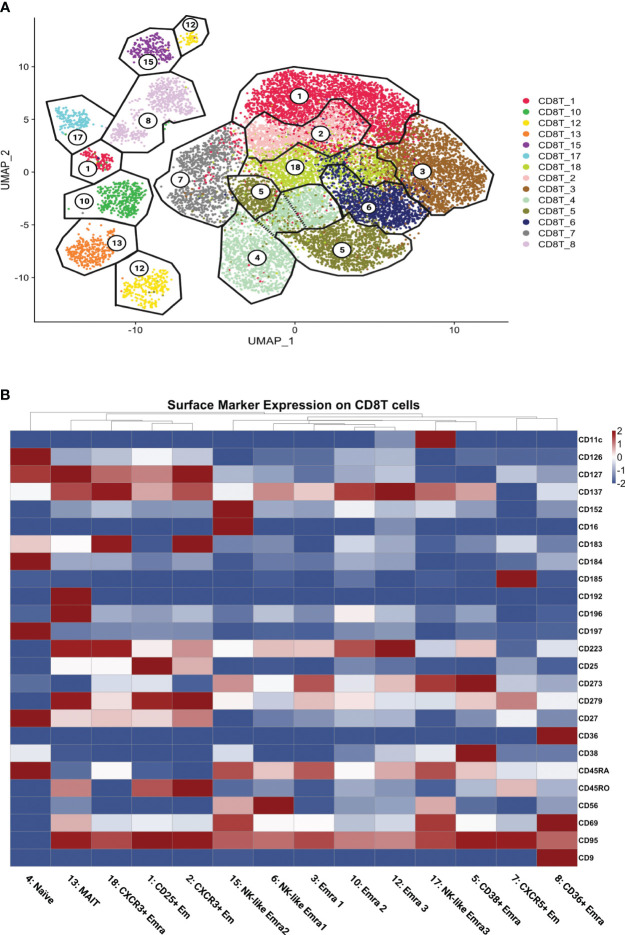
CD8+ T cell clustering and naming. **(A)** UMAP showing CD8+ T cells clustered by the Louvain algorithm based on expression of non-negative surface markers. CD8+ T cells formed 14 clusters; gates (black lines) overlaid and cluster numbers indicated. **(B)** Scaled (log2) heatmaps of antibody expression for each CD8+ T cell cluster. Cluster names indicated. Mean expression: highest: dark red, lowest: dark blue.

Next, we asked whether these pathways were enriched in all CD8+ T cells or only in certain subsets. We used unsupervised Louvain clustering and displayed all CD8+ T cells as a Uniform Manifold Approximation and Projection for Dimension Reduction (UMAP) map. Based on the cell surface markers as measured by CITE-Seq, we initially identified 18 clusters, 4 of which were identified as likely doublets that were not considered further. Gates (analogous to standard flow cytometry workflow) were overlaid (**Figure 2A**). Cell counts are shown in **Table S6**.

All 14 singlet CD8+ T cell clusters were called based on well-established cell surface markers (**Figure 2B**; **Supplementary Excel Sheet 1**). The main subsets of CD8 T cells were effector memory (Em) and terminally differentiated effector memory (Emra) CD8 T cells. CD45RA+ CCR7- cells are considered terminally differentiated effector memory cells (TEMRA/Emra), these CD45RA re-expressing cells are generally assumed to be cytolytic with low proliferative potential (25). Effector memory (Em) subsets were CD45RA low and CD45RO high (26).

### Transcriptomes

**Figure 3:**
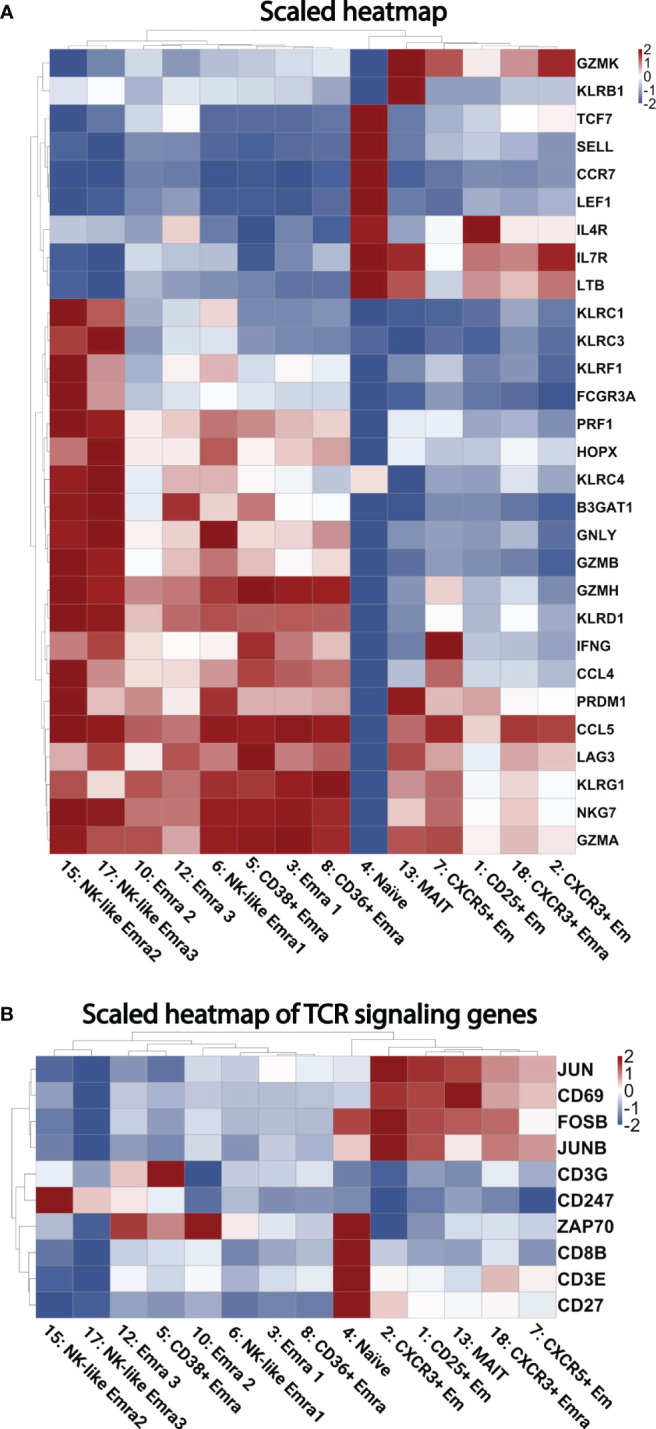
Heatmaps of gene expression. **(A)** Scaled heatmap of selected differentially expressed genes (filtered based on adjusted p-value < 0.05, avg_log2FC > 0, and pct.1 (percent of cells expressing each gene in each cluster)/pct.2 (percent of cells expressing each gene in all other clusters) >2.5 in each subcluster. **(B)** Scaled heatmap of T cell receptor activating genes.

Next, we analyzed the transcriptomes of each single cell. The gene expression in each cluster was tested against the other CD8+ T cell clusters. (**Figure S2**). **Figure 3A** shows the heatmap of selected cytotoxic, cytolytic genes and chemokines. The transcriptomes confirmed the identity of the clusters, showed different cluster signatures, and expanded phenotypic information. The uniquely enriched genes is listed in **Table S7**.

Naïve-determining genes such as TCF7, SELL, CCR7, and LEF1 were enriched in cluster 4. High GZMK levels were seen in Em clusters 2 and 7; this cytolytic marker has been described as a hallmark of inflammaging (27). Clusters 3, 5, 10 and 12 were confirmed to be EMRA populations by expression of GZMB, PRF1, NKG7, KLRD1, KLRG1, and lack of CD27 (11). A heatmap of TCR signaling genes is shown in **Figure 3B**, showing activation within several clusters such as the CD25+ Em cluster, and several EMRA clusters.

The combination of high GNLY, KLRC3, KLRF1, KLRC1, and KLRC4 further defined 15 and 17 as cytotoxic CD8 EMRA clusters (28). Cluster 13 showed high KLRB1 (which encodes CD161) and IL-7R expression (encodes CD127). These features are consistent with mucosal associated invariant T cells (MAIT) (29). The significantly differentially expressed genes that define each cluster are listed in **Supplementary Excel Sheet 2**.

### Pathways in CD8+ T cell clusters

**Figure 4:**
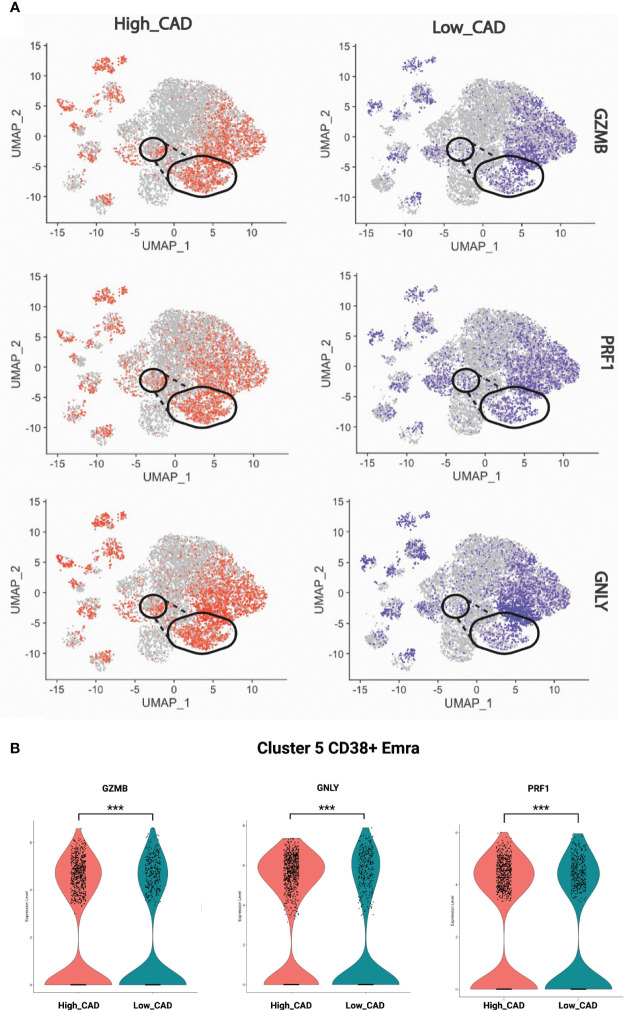
Expression of selected cytotoxic genes. **(A)** GZMB, PRF1, and GNLY projected onto CD8+ T cell UMAPs. CAD high (red) and CAD low (blue) projected to UMAPs from all the samples. Gate of cluster 5 overlaid. **(B)** Violin plots showing the proportion of GZMB, PRF1, and GNLY in CAD high vs CAD low. The significance level was determined using Wilcoxon test, *** indicates a significance value of < 0.001.

Next, we projected the TCR signaling, cytotoxic and exhaustion-relevant genes onto the CD8+ T cell UMAP. The TCR related genes were chosen based on the genes enriched in the TCR pathways (**Tables S4, S5**); accordingly, GZMB, PRF1, and GNLY localized to several Em and Emra clusters in cases and controls. More cells in cluster 5 expressed these genes in CAD+ than CAD- samples (**Figure 4A**). **Figure 4B** displays the significantly higher proportion of GZMB, PRF1, and GNLY in cluster 5 (CD38+ Emra). This finding indicates that CD38+ EMRA are enriched in TCR and cytotoxic signaling.

### Differentially expressed genes in clusters

**Figure 5:**
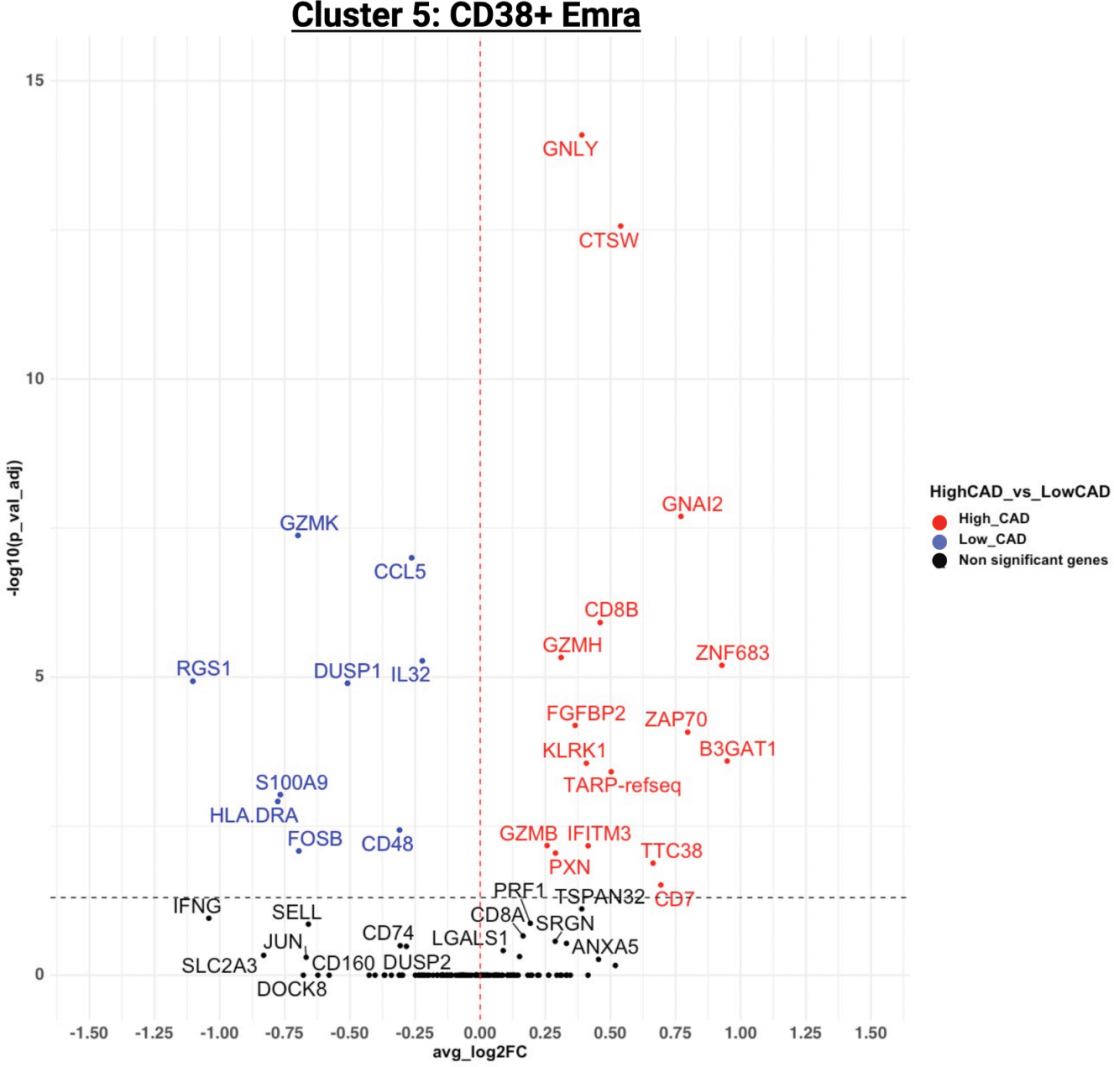
Volcano plot of the differentially expressed genes (DEGs) for cluster 5 in CAD high (red) vs CAD low (blue). For genes/markers to be differentially expressed (DE), the threshold was set to 0 for the average log_2_ fold change and filtered for an adjusted p-value of < 0.05.

**Figure 6:**
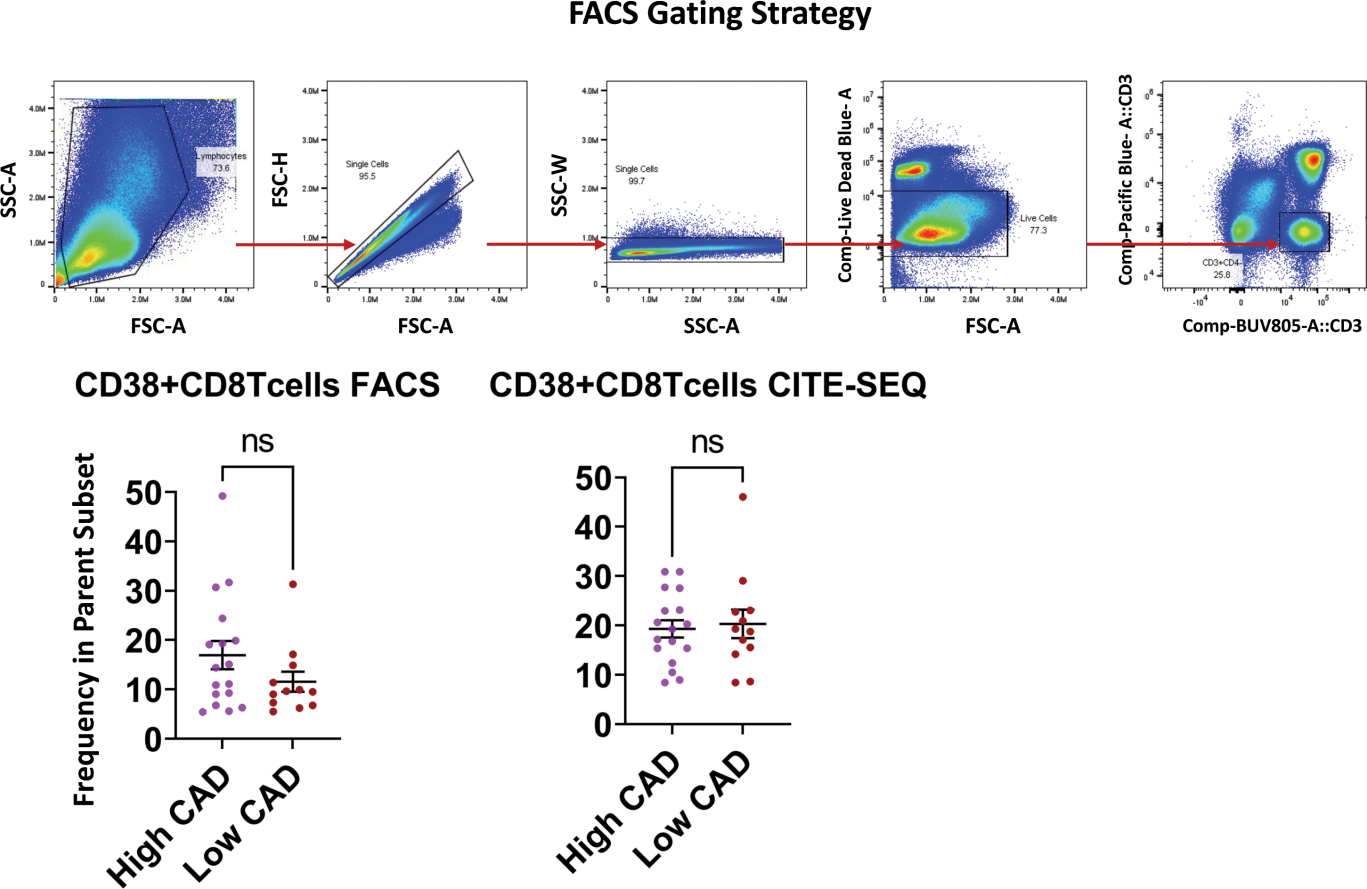
Validation of CD38 expression by flow cytometry and CITE-SEQ. PBMC samples from the same 29 patients with or without CAD or diabetes were analyzed by FACS and CITE-seq for the indicated markers. The gating strategy for morphology, singlets, live CD3+CD4- T cells is shown as dot plots. Frequencies of CD38+ expressing cells were analyzed within the parent CD3+CD4- gate. Comparison of CD38 expressing CD8+Tcells between high CAD and low CAD respectively. Results are represented as mean ± SEM. Paired t test. ns, not significant.

**Figure 5** shows the significantly regulated genes within cluster 5 (CD38+ Emra) in cases and controls. CD38 expression is associated with T cell activation, and microscopic studies have reported its localization to the immune synapse and close proximity with the T cell receptor upon activation (30). Studies have suggested the potential use of CD38 as a therapeutic target in cardiac disease (31). CD38 expression was confirmed by FACS in CD8 T cells (CD3+ CD4- CD19-) in 29 patients for whom second tubes were available (of the 61 pateints interrogated by CITE-Seq) (**Figure 6**). 14.7 ± 1.9% expressed CD38 by FACS. By CITE-Seq, 19.7 ± 1.6% expressed CD38 (mean ± SEM). There was no significant difference between CAD-hi and CAD-lo patients. (**Supplemental Figure S6**). Several cytotoxic genes including GNLY, GZMH, GZMB, KLRK1 and CTSW were significantly upregulated in cluster 5 CD8 T cells of CAD+ subjects. **Tables S9A-L** lists the up- and down-regulated genes within all remaining clusters.

## Discussion

CD8+ T cells in subjects with CAD show highly significant enrichment for genes encoding components of the TCR signaling pathway. This is consistent with recent data suggesting that atherosclerosis is associated with a break of tolerance to self-antigens. Chowdhury et al. (10), reported 2 populations of CD8 T Em cells in the plaques of patients with CAD. Consistent with our data, one subset of plaque CD8 T cells that was more prominent in complex plaques expressed a proinflammatory and cytolytic signature. They suggested that this proinflammatory state could be considered the cause of the weakening of the fibrous cap and subsequent plaque instability and rupture. The same group also reported TCR signaling pathway enrichment in plaque. We confirm these findings and extend them to a much larger patient population (60 subjects). We identified comparable gene expressions between cluster 7 (CXCR5+ Em) in our paper and the CD8 CTL Tem1 cluster reported by (10), which was found at high levels in the plaques of CAD patients. The second CD8+ cluster described by these authors (CD8 CTL Tem2), which tracked with plaque progression, had similar gene expression as our 2 cytotoxic CD8 clusters (clusters 15&17). Similarly, Depuydt et al. (11), subclustered CD8 T cells into 11 clusters. Their EMRA cluster 1 had a similar profile to our cytotoxic EMRA 1 cluster (cluster 15), while their EMRA cluster 4 had a similar gene profile to another cytotoxic EMRA 2 cluster (cluster 17).

PBMCs are attractive for scRNA-Seq studies because they are available in many clinical studies of specific populations with defined diseases and outcomes. scRNA-Seq has been applied to human PBMCs in atherosclerosis (6, 32). Fernandez et al., studied surface marker expression using CITE-Seq, but this approach was only applied to one subject (6). A key advantage of our approach is that CD8 T cells are identified by surface markers as CD3+CD4-CD8+CD19-, the same way one would gate in flow cytometry. This circumvents the problem of dropouts in scRNA-Seq (33). For example, in 8% of the 16,352 CD8 T cells studied here, CD8 transcript was not detected, although it must have been present, because the CD8 protein was expressed on the surface. A second advantage is that clustering is done based on thresholded antibodies, greatly reducing the noise, and focusing on the antibody signal (16, 34, 35). Using only antibodies for clustering sharpens the detection of gene expression differences, because the gene expression does not affect the clustering.

This study is the largest scRNA-Seq and CITE-Seq study of CD8 T cells from subjects with and without angiographically verified CAD. Thus, the CAD status of each patient is definitive and direct. Weaknesses include a relative paucity of women (16 women vs 44 men) and the narrow race and ethnicity composition (almost all subjects were Caucasian).

In conclusion, we show that CD8 T cells from CAD+ subjects show clear differences in gene expression. The most important finding is that TCR signaling and cytotoxic pathways were upregulated in subjects with CAD. Expression of several of these TCR signaling genes were important CAD predicting genes and localized to a CD38+ Emra cluster. Altogether these findings suggest that CD8+ T cells in subjects with CAD are continuously or repeatedly exposed to atherosclerosis antigens and can kill target cells.

## Materials and methods

### Human subjects

Subjects ranging in age from 40-80 with suspected CAD from the Coronary Assessment in Virginia (CAVA) cohort were recruited for the study through the Cardiac Catheterization laboratory at the University of Virginia Health System. Prior to enrollment, written consent was obtained from all participants. The study received approval from the Human Institutional Review Board (IRB no.15328). Peripheral blood samples were collected before the subjects underwent catheterization.

### Quantitative coronary angiography

Standard cardiac catheterization was conducted, with two orthogonal views of the right coronary artery and four of the left coronary artery, in accordance with accepted standards. Quantitative Coronary Angiography (QCA) was done at the end-diastolic frame using automatic edge detection. For each lesion, the frame was selected based on demonstration of the most severe stenosis with minimal foreshortening and branch overlap. The minimum lumen diameter, reference diameter, percent diameter stenosis, and length of stenosis were calculated. Experienced blinded investigators carried out the analysis. The Gensini scoring system (36) was used to assign a score of disease severity to each patient. Each arterial segment is assigned a score of 0-32 based on the degree of stenosis. The severity score for each segment was multiplied by 0.5-5, depending on the location stenosis. Scores for all segments were then added together to give a final score of angiographic disease burden. The study did not perform adjustment for collateral circulation. Subjects with a Gensini score exceeding 30 were categorized as having high CAD severity, those with a Gensini score below 6 were classified as low CAD severity subjects (controls).

### Preparation of PBMC samples for CITE-seq

Within the first hour of collection, blood from cases and controls was drawn into BD K2 EDTA vacutainer tubes and processed at room temperature. Ficoll-Paque PLUS (GE Healthcare Biosciences AB) gradient centrifugation using SepMate-50 tubes (Stemcell Technologies Inc) was used to isolate PBMCs, following the manufacturers protocol. Live cell counts were quantified by Trypan blue staining of PBMCs. PBMCs were cryopreserved in freezing solution (90% FBS with 10% DMSO) and the vials were stored in Mr. Frosty (Thermo Fisher) for 48 hours at -80°C and were then stored in liquid nitrogen until used. To avoid batch effects, 8 samples were processed each day, thawed in a 37°C-water bath, centrifuged at 400 xg for 5 minutes, and pellets resuspended in cold staining buffer. The BD Rhapsody scanner was used to evaluate the viability and cell count of each tube (**Table S2**). **Table S3** shows all the reagents, manufacturers, and catalogue numbers. The tubes were then centrifuged at 400 xg for 5 minutes and resuspended in a cocktail of 49 AbSeq antibodies (2 μL each and 20 μL of staining buffer (SB), listed in **Table S3**) on ice for 30-60 minutes per the manufacturer’s recommendations, washed and counted again. Of the 65 samples investigated, 60 passed quality control (cell viability > 75%). Cells from each subject were sample tagged using a Sample Multiplexing Kit (BD Biosciences) which contains oligonucleotide cell labeling, washed 3x, mixed, counted, stained with the 49-antibody mix, washed 3 times again and loaded onto Rhapsody nanowell plates (4 samples per plate).

### Library preparation

In addition to viability, pre-sequencing quality control measures (QC) included Agilent TapeStation using high sensitivity D1000 screentape. Each tube was then cleaned with AMPure XP beads and washed in 80% ethanol. The cDNA was eluted, a second Tapestation QC was performed, and diluted as needed. The samples were pooled and sequenced as recommended: CITE-Seq: 40,000 reads per cell, mRNA: 20,000 reads per cell, sample tags: 600 reads per cell on Illumina NovaSeq using S1 and S2 100 cycle kits (Illumina) (67x8x50 bp). FASTA and FASTQ files were uploaded to the Seven Bridges Genomics pipeline, where the data was filtered to generate matrices and CSV files. This analysis generated draft transcriptomes and surface phenotypes of 213,515 cells (496 genes, 49 antibodies). After removing multiplets based on sample tags and undetermined cells, 175,628 cells remained. Doublet Finder (https://github.com/chris-mcginnis-ucsf/DoubletFinder) was used to remove additional doublets, leaving 162,454 cells. 306 CD8+ T cells were removed because they looked like biological doublets with myeloid cells (37). CD8+ T cells were defined as CD19- CD14- CD16- CD3+ CD4- CD8+. All antibody data were CLR (centered log-ratio) normalized and converted to log_2_ scale. All transcripts were normalized by total UMIs of a gene across all cells, scaled to 1 million, and converted to a log2 scale.

### Thresholding

Combined protein and transcript panel single cell sequencing can lead to background noise. This is partly due to non-specific binding contributing to the antibody signal, because the Fc receptor block is incomplete. Another source of background is due to unbound oligonucleotide-tagged antibody that remain in the nanowell getting amplified and subsequently sequenced. To address this each antibody was assigned a threshold by determining its signal in a known negative cell or by deconvolution of overlapping normal distributions (the function ‘normalmixEM’ was used to deconvolute the overlapping distributions in the package ‘mixtools’). To account for all sources of background noise ridgeline plots for each antibody in each main cell type was used to set the best threshold. Ridgeline plots for CD8 T cells, CD4 T cells, B cells, NK, and myeloid cells as we described previously, were constructed (38) (39)., This yielded thresholds for 30 markers used (**Table S8**).

### Clustering

We reclustered CD8 T cells by Uniform Manifold Approximation and Projection (UMAP) dimensionality reduction to project the cells onto the 2D space. UMAP was the algorithm chosen because it preserves the global data structure while capturing local similarities. We ran the algorithm over the first 20 principal components provided by Harmony. The Standard Louvain clustering algorithm was used to cluster the data. The parameter resolution was set to 0.15 and random seed set to 42 to ensure the results could be reproducible. Before running the Louvain clustering, we filtered out non-expressed antibodies, including CD19 and CD4 for CD8+ T cells, and antibodies where thresholds could not be determined.

### Comparing gene expression

We identified the differentially expressed genes/surface markers (DEGs) by using the “FindAllMarkers: function on Serurat. For genes/markers to be differentially expressed (DE), the threshold was set to 0 for the average log_2_ fold change and filtered for an adjusted p-value of < 0.05. DE genes were visualized by using the “EnhancedVolcano” function of the EnhancedVolcano R package.

### Random forest model

To identify genes with the highest capability of distinguishing between disease groups a machine learning (ML) approach was implemented. A random forest classifier with permutation feature importance was trained on normalized gene expression data (40). Random Forest is an ensemble learning algorithm in which the best parameter at each node is made from a randomly selected number of features. The “permutation feature importance” is the reduction in the performance of a model upon random shuffling of a single feature value. This procedure disrupts the association between the feature and the target class, thereby indicating the extent to which the model relies on the features based on the observed decrease in the model score. This method exhibits the advantage of being independent of the specific model used, allowing for multiple calculations with various permutations of the feature. To perform hyperparameter optimization during permutations and avoid overfitting, a 3-fold cross validation with GridSearch was used. The resultant “importance scores” were scaled to the 0-100 range for a better comparison. A higher score means that the gene is more important for classifying the groups (CAD cases vs controls).

### Pathway analysis

Gene ontology pathway analysis of the genes preferentially upregulated in different disease states using the Enrichr pathway analysis tool was used.

### Flow cytometry

PBMCs were isolated, thawed and counted as described above. 10ml 1XPBS was added to each sample, followed by centrifugation at 400 g for 10 minutes at RT. 1.5X10^6^ cells were seeded into 96 well plates, incubated with Human TruStain FcX™ (Fc Receptor Blocking Solution; Biolegend; Catalogue no: 422302) for 10min at 4°C followed by fixable viability dye (Live Dead Blue; Invitrogen cat. No: L34962) for 30min at 4°C, washed, and the antibody master mix was were added for 40min at 4°C: anti-CD3 BUV805 (BD Biosciences; Catalogue no: 612895; Clone UCHT1) anti-CD4 PB (Biolegend; Catalogue no: 344620; Clone SK3), anti-CD38 APC (Biolegend; Catalogue no: 356606; Clone HB-7),CD11c Percp (Biolegend; Catalogue no: 337234; clone Bu15), CD14 APC-cy7 (Biolegend; Catalogue no: 325620; clone HCD14), CD16 BV785 (Biolegend; Catalogue no: 302046; clone 3G8), CD19 PE/Fire 700 (Biolegend; Catalogue no: 302276; clone HIB19), CD56 BV570 (Biolegend; Catalogue no: 362540; clone 5.1H11), CD11b BV605 (Biolegend; Catalogue no: 301332; clone ICRF44), HLA-DR BV650 (Biolegend; Catalogue no: 307650; clone L243), CD192 (CCR2) PE (Biolegend; Catalogue no: 357206; clone K036C2), CD33 Percpcy5.5 (Biolegend; Catalogue no: 303414; clone WM53), CD196 (CCR6) PE/Fire 640 (Biolegend; Catalogue no: 353449; clone G034E3), CD163 PE/Dazzle 594 (Biolegend; Catalogue no: 333624; clone GHI/61), CD69 AF700 (Biolegend; Catalogue no: 310922; clone FN50), CD86 AF647 (Biolegend; Catalogue no: 305416; clone IT2.2), CD43 PE-cy7 (Biolegend; Catalogue no: 343208; clone CD43-10G7), CD274 (PD-L1) BB515 (BD Biosciences; Catalogue no: 564554; clone MIH1), CD45RA BV510 (Biolegend; Catalogue no: 304142; clone HI100), CD95 PE-cy5 (Biolegend; Catalogue no: 305610; clone DX2), CD206 BV421 (Biolegend; Catalogue no: 321126; clone15-2), CD45RO BV711 (Biolegend; Catalogue no: 304236; clone UCHL1) and CD183 (CXCR3) BUV615 (BD Biosciences; Catalogue no: 751126; clone LS177-1C6). Data was acquired on a Cytek Aurora 5 laser Spectral Flow Cytometer (Cytek Biosciences, Fremont, CA, USA) with automatic compensation and analyzed with FlowJo software. Fluorescence-minus-one (FMO) controls from healthy donors were used to set the gates. **Supplemental Material**: includes information on the clinical characteristics of all the subjects in the cohort (**Tables S1A-B**), the viability of the samples taken (**Table S2**), details on antibodies used and the thresholds used (**Table S3**), **Tables S4**, **S5** shows the data from pathway analysis obtained using EnrichR and IPA shown in **Figure 1D** and **Figure S1**, respectively. Cell counts of the CD8+ T cells clustered is shown in **Table S6**. **Table S7** provides a brief description of all the genes shown in the heatmap in **Figure 3A**. **Tables S9A-L** provides data on the differentially expressed genes based on disease status within all clusters.

## Data availability statement

The datasets presented in this study can be found in online repositories. The names of the repository/repositories and accession number(s) can be found below: GSE190570 (GEO).

## Ethics statement

The study received approval from the Human Institutional Review Board (IRB no.15328). The studies were conducted in accordance with the local legislation and institutional requirements. The human samples used in this study were acquired from primarily isolated as part of your previous study for which ethical approval was obtained. Written informed consent for participation was not required from the participants or the participants’ legal guardians/next of kin in accordance with the national legislation and institutional requirements.

## Author contributions

CH and KL designed the study. CM collected samples and data for the angiography analysis for calculating the score. RS and SI analyzed clinical data. RS and CD ran the scRNA-Seq experiments. AK, MO, SS, YG, AA, and KL analyzed the data. AK conducted the bioinformatics analysis. SI and KL wrote the manuscript. All authors contributed to the article and approved the submitted version.

## Supplementary material

SUPPLEMENTARY DATA SHEET 2 Differential expression of antibodies by CITE-Seq comparing one vs. all clusters. Sheet 1 contains a list of differentially expressed antibodies for all CD8 T cells, organized by cluster. The other sheets contain data for each CD8 T cell cluster as named in the tab. p_val is the raw (unadjusted) p value. avg_log2FC is the average log fold change where positive values indicate higher expression in that cluster compared to others. pct.1 is the percent of cells expressing that gene in the that particular cluster. pct.2 is the percent of cells that express that gene in the rest of the clusters. p_val_adj is the p value adjusted for multiple comparisons. Cluster identifies the cluster with significant differences. Gene is the name of each antigen.

SUPPLEMENTARY DATA SHEET 3 Gene expression by scRNA-Seq comparing one vs. all clusters. Sheet 2 contains data for all CD8 T cells, organized by cluster. The other sheets contain data for each CD8 T cell cluster as named in the tab. p_val is the raw (unadjusted) p value. avg_log2FC is the average log fold change where positive values indicate higher expression in cases than controls. pct.1 is the percent of cells expressing that gene in cases. pct.2 is the percent of cells that express that gene in controls. p_val_adj is the p value adjusted for multiple comparisons. cluster identifies the cluster with significant differences. gene is the gene name.
