# Supplementary material for: Single cell transcriptomics reveals recent CD8T cell receptor signaling in patients with coronary artery disease
Source: Front Immunol. 2023 Sep 27;14:1239148. doi: 10.3389/fimmu.2023.1239148 (PMC10565000; doi:10.3389/fimmu.2023.1239148)
Supplement: Supplementary file 3 [file DataSheet_1.docx]

**SUPPLEMENTAL MATERIAL**

**Single Cell Transcriptomics Reveals Increased CD8T Cell Receptor Signaling in Patients with Coronary Artery Disease**

Shahad Iqneibi^1^, Ryosuke Saigusa^2^, Amir Khan^1^, Mohammad Oliaeimotlagh^1^, Sujit Silas Armstrong Suthahar^2^, Sunil Kumar^1^, Rishab Gulati^2^, Vasantika Suryawanshi^2^, Ahmad Alimadadi^2^, William Pandori^2^, Jeff Makings^2^, Christopher P. Durant^3^, Yanal Ghosheh^2^, Fabrizio Drago^3^, Coleen A. McNamara,^3^ Avishai Shemesh^4^, Lewis L. Lanier^4^, Catherine C. Hedrick^1,2^, Klaus Ley^1,2,5^

^1^ Immunology Center of Georgia, Augusta University, Augusta, GA 30912, USA

^2^La Jolla Institute for Immunology, La Jolla, CA, USA.

^3^Cardiovascular Research Center, Cardiovascular Division, Department of Medicine, University of Virginia, Charlottesville.

^4^Parker Institute for Cancer Immunotherapy, University of California, San Francisco, CA, USA; Department of Microbiology and Immunology, University of California, San Francisco, CA, USA.

^5^Department of Physiology, Augusta University, Augusta, GA 30912, USA

*Corresponding Author

Klaus Ley, MD

Co-Director, Immunology Center of Georgia (IMMCG)

1410 Laney Walker Blvd, CN4315

Augusta GA, 30912, USA

(858) 472-7253 (tel)

(706) 446-0296 (fax)

[kley@augusta.edu](mailto:kley@augusta.edu)


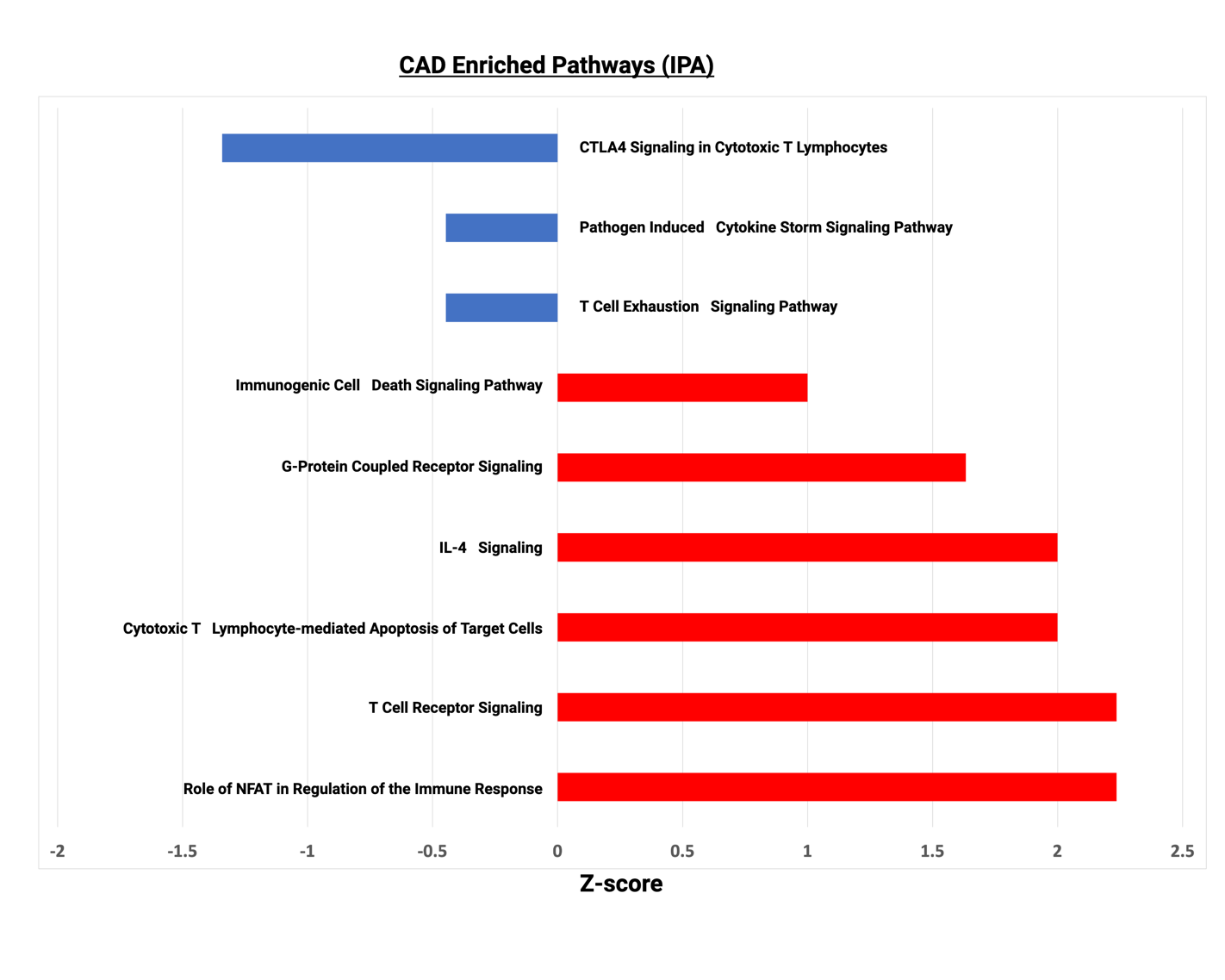


**Figure S1:** Ingenuity Pathway Analysis (IPA) showing significantly enriched pathways (p-value < 0.05) of CAD high vs CAD low. Red bars represent a positive z score, and blue bars indicate a negative z score. Refer to table S5 for additional information.


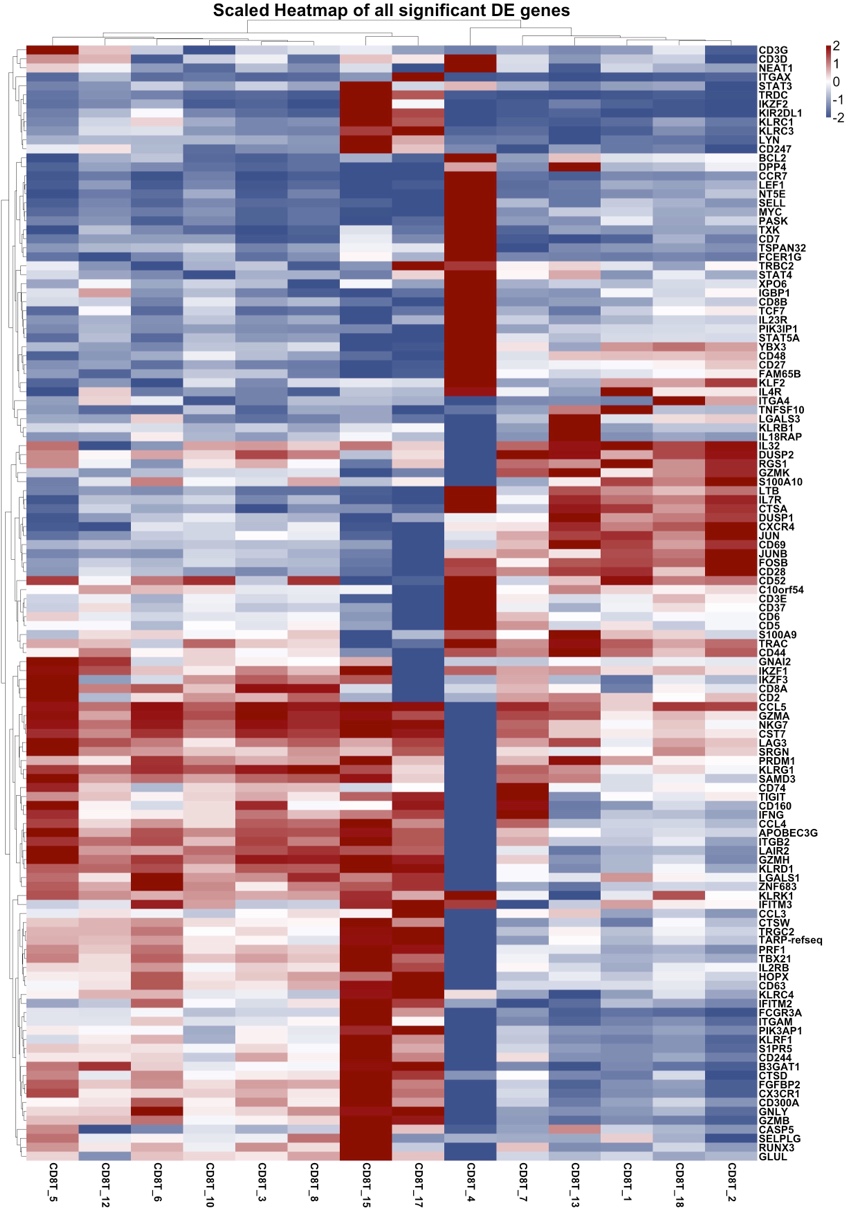


**Figure S2:** Scaled heatmap of all differentially expressed genes (filtered based on an adjusted p value of <0.05, avg_log2FC >0, and pct.1 (percent of cells expressing each gene in each cluster)/pct.2 (percent of cells expressing each gene in all other clusters) > 2.5 in each subcluster.


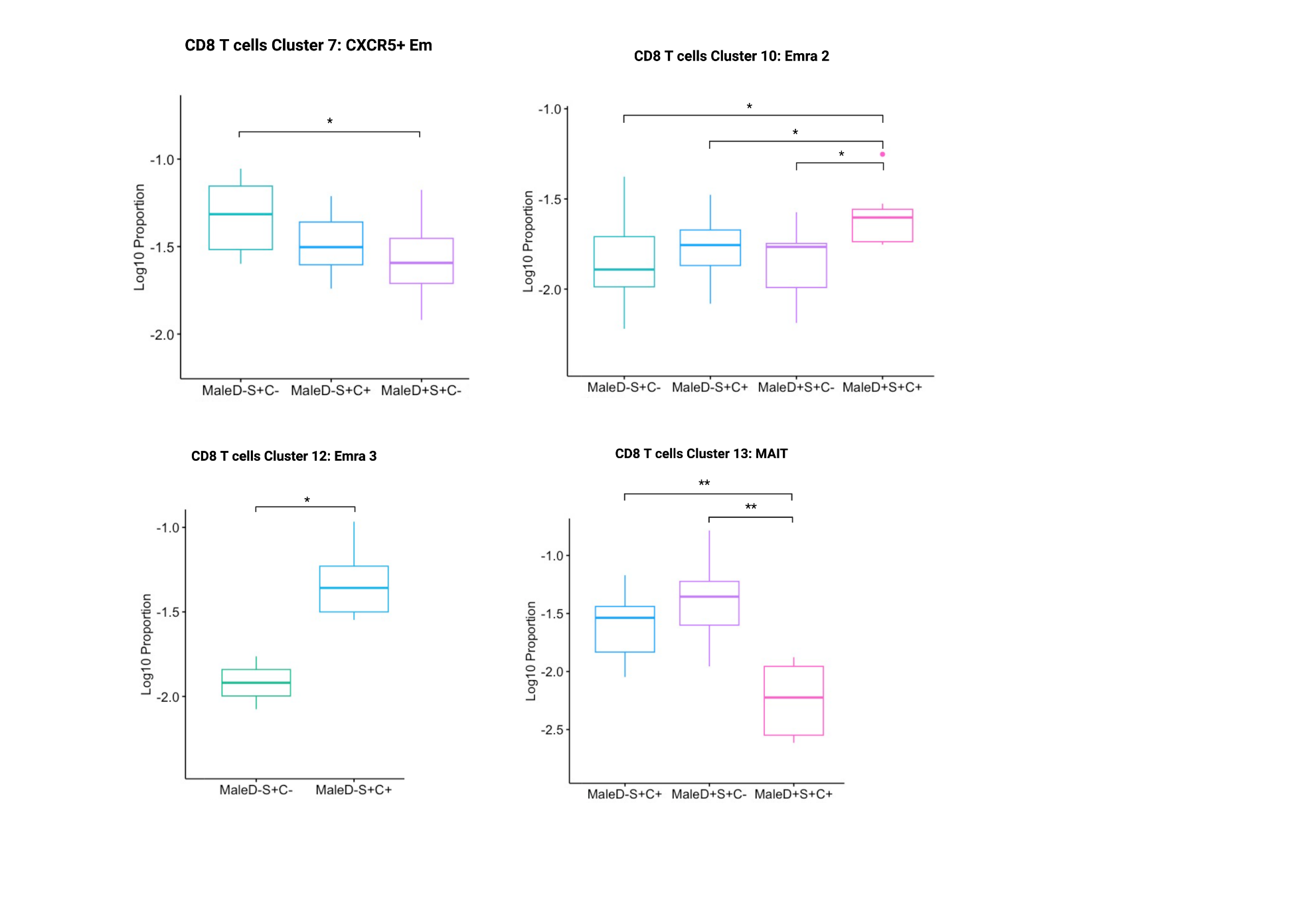


**Figure S3:** To see if DM, CVD, and sex correlated with the abundance (cell number) of CD8 T cell subsets, we compared the log odds ratios of CD8 T cell cluster for each clinical variable, in order make the analysis independent of the absolute number of cells per patient. Only 4 clusters showed significant differences, cluster 7 (CXCR5+ Em) was reduced in diabetic males without CAD compared to controls. The proportion Emra 2 (cluster 10) was significantly (p < 0.05) higher in diabetic, statin treated men with CAD compared to the matched CAD- DM+ S+ group. The proportion of this cluster was also significantly higher in the matched CAD group that were statin treated with DM vs without. Cluster 12 (Emra 3) similarly was higher in CAD+ DM- statin treated males then in CAD-. Cluster 13 (MAIT) was significantly reduced in statin-treated men with diabetes and CAD vs DM- and CAD-.

**Table S1A and B. Clinical characteristics of all the patients**

1. **CAD Low vs CAD High in all the participants**

| Variable  Count [%] or Mean [± SD] | **CAD Low (n=29)** | **CAD High (n=31)** | **p-value** |
| --- | --- | --- | --- |
| **Demographics** |  |  |  |
| Age (years) | 64 [±8.27] | 65 [±9.12] | 0.41 |
| Sex (Male) | 22 (76%) | 22 (71%) | 0.54 |
| Race (Caucasian) | 27 (93%) | 31 (100%) | 0.50 |
| Ethnicity (Non-Hispanic) | 27 (93%) | 30 (97%) | 0.92 |
| Diabetes (Yes) | 13 (45%) | 14 (45%) | 0.93 |
| Smoking | 18 (62%) | 16 (52%) | 0.34 |
| BMI | 34.2 [±6.65] | 30.6 [±6.02] | 0.04 |
| BP Systolic | 135 [±19.85] | 143 [±19.36] | 0.09 |
| BP Diastolic | 78 [±12.56] | 76 [±13.97] | 0.57 |
| **Medications** |  |  |  |
| Statins (Yes) | 24 (83%) | 26 (84%) | 0.88 |
| Diuretics (Yes) | 4 (14%) | 11 (35%) | 0.06 |
| Beta Blockers (Yes) | 18 (62%) | 16 (52%) | 0.34 |
| Calcium Channel Blockers (Yes) | 4 (14%) | 9 (29%) | 0.17 |
| ACE (Yes) | 11 (38%) | 11 (35%) | 0.77 |
| ATR (Yes) | 4 (14%) | 2 (6%) | 0.32 |
| NSAID (Yes) | 23 (79%) | 28 (90%) | 0.39 |
| **Lab Values** |  |  |  |
| Creatinine | 0.81 [±0.25] | 0.84 [±0.20] | 0.84 |
| Hs-CRP | 3.3 [±3.71] | 2.64 [±2.48] | 0.78 |
| Total Cholesterol (mg/dL) | 141 [±27.01] | 152 [±46.99] | 0.65 |
| Triglyceride (mg/dL) | 137 [±76.39] | 114 [±67.66] | 0.19 |
| HDL Cholesterol (mg/dL) | 40 [±11.05] | 44 [±14.73] | 0.36 |
| LDL Cholesterol (mg/dL) | 79 [±23.49] | 89 [±38.98] | 0.60 |
| Glucose (mg/dL) | 121 [±44.74] | 113 [±29.46] | 0.98 |
| A1c (%) | 6.6 [±1.56] | 6.2 [±0.98] | 0.81 |
| **Disease Severity** |  |  |  |
| Gensini Scores | 2.3 [±2.46] | 61.3 [±31.66] | <.0001 |

1. **Men vs Women in all the participants**

| Variable  Count [%] or Mean [± SD] | Men (n=44) | Women (n=16) | p-value |
| --- | --- | --- | --- |
| **Demographics** |  |  |  |
| Age (years) | 64 [±8.86] | 67 [±8.04] | 0.24 |
| Race (Caucasian) | 41 (93%) | 16 (100%) | 0.27 |
| Ethnicity (Non-Hispanic) | 41 (93%) | 16 (100%) | 0.89 |
| Diabetes (Yes) | 17 (39%) | 10 (62%) | 0.15 |
| Smoking (Yes) | 23 (52%) | 11 (69%) | 0.38 |
| BMI | 32.9 [±6.33] | 33.4 [±7.10] | 0.37 |
| BP Systolic | 138 [±16.86] | 141 [±26.53] | 0.91 |
| BP Diastolic | 78 [±13.44] | 75 [±12.72] | 0.30 |
| **Medications** |  |  |  |
| Statins (Yes) | 34 (77%) | 16 (100%) | 0.13 |
| Diuretics (Yes) | 8 (18%) | 7 (44%) | 0.06 |
| Beta Blockers (Yes) | 24 (55%) | 10 (62%) | 0.76 |
| Calcium Channel Blockers (Yes) | 6 (14%) | 7 (44%) | 0.02 |
| ACE (Yes) | 14 (32%) | 8 (50%) | 0.27 |
| ATR (Yes) | 5 (11%) | 1 (6%) | 0.52 |
| NSAID (Yes) | 38 (86%) | 13 (81%) | 0.35 |
| **Lab Values** |  |  |  |
| Creatinine | 0.85 [±0.24] | 0.75 [±0.15] | 0.04 |
| Hs-CRP | 2.4 [±2.82] | 4.4 [±3.44] | 0.001 |
| Total Cholesterol (mg/dL) | 140 [±35.70] | 164 [±42.58] | 0.05 |
| Triglyceride (mg/dL) | 123 [±74.17] | 131 [±68.80] | 0.54 |
| HDL Cholesterol (mg/dL) | 39 [±10.68] | 48 [±16.93] | 0.06 |
| LDL Cholesterol (mg/dL) | 81 [±31.30] | 94 [±35.20] | 0.16 |
| Glucose (mg/dL) | 116 [±39.98] | 118 [±30.93] | 0.41 |
| A1c (%) | 6.3 [±1.21] | 6.8 [±1.45] | 0.06 |
| **Disease Severity** |  |  |  |
| Gensini Scores | 36.8 [±42.13] | 24.1 [±19.05] | 0.58 |

**Table S2. The viability of each sample tube**

| **Sample #** | **Average** | **Sample #** | **Average** |
| --- | --- | --- | --- |
| 1 | 92.50% | 33 | 77.30% |
| 2 | 90.00% | 34 | 91.38% |
| 3 | 84.00% | 35 | 90.79% |
| 4 | 89.00% | 36 | 87.86% |
| 5 | 93.00% | 37 | 84.09% |
| 6 | 90.50% | 38 | 90.06% |
| 7 | 93.00% | 39 | 90.09% |
| 8 | 90.00% | 40 | 95.53% |
| 9 | 94.79% | 41 | 93.23% |
| 10 | 91.75% | 42 | 87.69% |
| 11 | 93.10% | 43 | 97.40% |
| 12 | 94.47% | 44 | 95.17% |
| 13 | 89.55% | 45 | 84.85% |
| 14 | 84.97% | 46 | 88.61% |
| 15 | 92.13% | 47 | 92.62% |
| 16 | 93.62% | 48 | 92.39% |
| 17 | 90.33% | 49 | 89.20% |
| 18 | 90.72% | 50 | 94.69% |
| 19 | 93.14% | 51 | 89.34% |
| 20 | 91.92% | 52 | 93.21% |
| 21 | 93.43% | 53 | 90.28% |
| 22 | 90.45% | 54 | 93.56% |
| 23 | 94.54% | 55 | 98.80% |
| 24 | 90.02% | 56 | 95.65% |
| 25 | 88.50% | 57 | 94.42% |
| 26 | 82.70% | 58 | 93.10% |
| 27 | 92.47% | 59 | 93.61% |
| 28 | 89.52% | 60 | 95.96% |
| 29 | 88.70% | **Min** | **77.1%** |
| 30 | 89.74% | **Max** | **98.8%** |
| 31 | 77.10% | **Median** | **91.09%** |
| 32 | 89.26% | **Average** | **90.83%** |

**Table S3. The information of 51 AbSeq antibodies**

| **Specificity** | | **Clone** 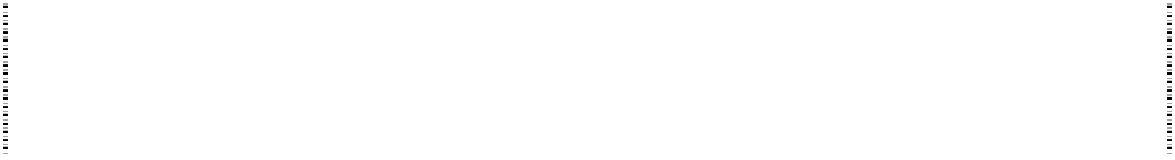 | | **Catalogue Number** | |
| --- | --- | --- | --- | --- | --- |
| CD11b | | M1/70 | | 940008 | |
| CD11c | | B-LY6 | | 940024 | |
| CD123 (IL-3RA) | | 7G3 | | 940020 | |
| CD126 (IL-6R) | | M5 | | 940090 | |
| CD127 (IL-7R) | | HIL-7R-M21 | | 940012 | |
| CD137 | | 4B4-1 | | 940055 | |
| CD14 | | MPHIP9 | | 940005 | |
| CD141 | | 1A4 | | 940079 | |
| CD142 | | HTF-1 | | 940280 | |
| CD152 (CTLA-4) | | BNI3 | | 940034 | |
| CD154 | | TRAP1 | | 940053 | |
| CD16 | | 3G8 | | 940006 | |
| CD163 | | GHI/61 | | 940058 | |
| CD183 (CXCR3) | | 1C6/CXCR3 | | 940030 | |
| CD184 (CXCR4) | | 12G5 | | 940056 | |
| CD185 (CXCR5) | | RF8B2 | | 940042 | |
| CD19 | | SJ25C1 | | 940004 | |
| CD192 (CCR2) | | 1D9 | | 940286 | |
| CD194 (CCR4) | | 1G1 | | 940047 | |
| CD195 (CCR5) | | 2D7/CCR5 | | 940050 | |
| CD196 (CCR6) | | 11A9 | | 940033 | |
| CD197 (CCR7) | | 3D12 | | 940014 | |
| CD2 | | RPA-2.10 | | 940046 | |
| CD20 | | 2H7 | | 940016 | |
| CD206 | | 19.2 | | 940068 | |
| CD223 (LAG-3) | | T47-530 | | 940080 | |
| CD25 | | 2A3 | | 940009 | |
| CD27 | | M-T271 | | 940018 | |
| CD3 | | SK7 | | 940000 | |
| CD36 | | CB38 (NL07) | | 940224 | |
| CD38 | | HIT2 | | 940013 | |
| CD4 | | SK3 | | 940001 | |
| CD45RA | | HI100 | | 940011 | |
| CD45RO | | UCHL1 | | 940022 | |
| CD56 |  | | NCAM16.2 | | 940007 |
| CD69 |  | | FN50 | | 940019 |
| CD8 |  | | RPA-T8 | | 940003 |
| CD86 |  | | 2331(FUN-1) | | 940025 |
| CD9 |  | | M-L13 | | 940078 |
| HLA-DR (CD74) |  | | G46-6 | | 940010 |
| CD24 |  | | ML5 | | 940028 |
| CD33 |  | | WM53 | | 940031 |
| IgM |  | | G20-127 | | 940276 |
| IgD |  | | IA6-2 | | 940026 |
| CD43 |  | | 1G10 | | 940728 |
| CD273 |  | | MIH18 | | 746072 |
| CD274 |  | | MIH1 | | 940035 |
| CD95 |  | | DX2 | | 940037 |
| CD279 |  | | MIH4 | | 940467 |
| TLR4-APC |  | | 610015 | | not applicable |
| SLAN-PE |  | | M-DC8 | | not applicable |
| PE |  | | E31-1459 | | 460077 |
| APC |  | | E30-221 | | 460078 |

**Table S4. Pathways enriched in CAD from Enrichr**

| **Pathway** | **P-value** | **Adjusted P-value** | **Odds Ratio** | **Combined Score** | **Genes** |
| --- | --- | --- | --- | --- | --- |
| Interleukin-2 signaling pathway | 1.39E-11 | 2.58E-09 | 14.99529682 | 374.863509 | **IFITM3;CX3CR1;SRGN;CD52;TCF7;ITGB2;PRF1;GZMB;NKG7;CTSW;KLRK1;GNLY;CD247;CTSD** |
| CTL mediated immune response against target cells | 2.40E-11 | 2.58E-09 | 377.9166667 | 9241.737782 | **ITGB2;PRF1;GZMB;CD3G;CD247** |
| Natural killer cell-mediated cytotoxicity | 6.41E-09 | 4.59E-07 | 34.44764268 | 649.8676624 | **ZAP70;FCGR3A;KLRK1;ITGB2;PRF1;GZMB;CD247** |
| CD8/T cell receptor downstream pathway | 1.80E-07 | 7.74E-06 | 47.85714286 | 743.2407523 | **PRF1;GZMB;CD3G;CD247;JUNB** |
| Lck and Fyn tyrosine kinases in initiation of T cell receptor activation | 1.79E-06 | 5.49E-05 | 171.0171429 | 2263.499882 | **ZAP70;CD3G;CD247** |
| T cell receptor signaling in naive CD8+ T cells | 3.52E-06 | 9.47E-05 | 45.93079585 | 576.7048055 | **ZAP70;PRF1;CD3G;CD247** |
| Inhibition of T cell receptor signaling by activated Csk | 6.00E-06 | 1.43E-04 | 106.8535714 | 1284.711594 | **ZAP70;CD3G;CD247** |
| PD-1 signaling | 2.74E-05 | 5.36E-04 | 61.02244898 | 641.0028653 | **ZAP70;CD3G;CD247** |
| MEF2D role in T cell apoptosis | 3.97E-05 | 7.11E-04 | 53.38392857 | 540.986612 | **ZAP70;CD3G;CD247** |
| T cell receptor regulation of apoptosis | 1.22E-04 | 0.001498819 | 7.337194198 | 66.09687176 | **IFITM3;ITGB2;GZMB;CD3G;JUNB;GNAI2** |
| Granzyme A-mediated apoptosis pathway | 2.29E-04 | 0.002464179 | 110.8444444 | 928.9653922 | **PRF1;GZMB** |
| Tob role in T-cell activation | 5.89E-04 | 0.004220762 | 65.17973856 | 484.7534602 | **CD3G;CD247** |
| T cell activation co-stimulatory signal | 7.22E-04 | 0.005004184 | 58.3128655 | 421.8429515 | **CD3G;CD247** |
| Mitochondrial pathway of apoptosis: BH3-only Bcl-2 family | 8.25E-04 | 0.005541832 | 18.11671733 | 128.6347137 | **GZMB;CTSW;CTSD** |
| T cell receptor signaling pathway | 0.002319491 | 0.012467266 | 12.49537815 | 75.80205374 | **ZAP70;CD3G;CD247** |
| Costimulation by the CD28 family | 0.008263045 | 0.034164515 | 15.78730159 | 75.71529959 | **CD3G;CD247** |
| T cell signal transduction | 0.010858117 | 0.044047078 | 13.63580247 | 61.67258521 | **ZAP70;CD3G** |

**Table S5: Pathways enriched in CAD from IPA**

| **Ingenuity Canonical Pathways** | **-log(p-value)** | **Ratio** | **z-score** | **Molecules** |
| --- | --- | --- | --- | --- |
| T Cell Receptor Signaling | 3.24 | 0.00966 | 2.236 | **CD247,CD3G,ITGB2,TCF7,TRGC2,ZAP70** |
| Role of NFAT in Regulation of the Immune Response | 2.12 | 0.00575 | 2.236 | **CD247,CD3G,FCGR3A/FCGR3B,GNAI2,TRGC2,ZAP70** |
| Cytotoxic T Lymphocyte-mediated Apoptosis of Target Cells | 3.15 | 0.0118 | 2 | **CD247,CD3G,GZMB,PRF1,TRGC2** |
| IL-4 Signaling | 1.79 | 0.0069 | 2 | **CD247,CD3G,GNAI2,TRGC2** |
| G-Protein Coupled Receptor Signaling | 2.93 | 0.0084 | 1.633 | **CCR7,CX3CR1,DUSP1,GNAI2,PXN,S1PR5** |
| Immunogenic Cell Death Signaling Pathway | 4.72 | 0.0426 | 1 | **GZMB,GZMH,GZMK,PRF1** |
| T Cell Exhaustion Signaling Pathway | 5.36 | 0.014 | -0.447 | **CD247,CD3G,GZMB,LAG3,PRDM1,TCF7,TRGC2,ZAP70** |
| Pathogen Induced Cytokine Storm Signaling Pathway | 3.39 | 0.0133 | -0.447 | **CCL5,GZMB,PRDM1,PRF1,SRGN** |
| CTLA4 Signaling in Cytotoxic T Lymphocytes | 2.45 | 0.00816 | -1.342 | **CD247,CD3G,ITGB2,TRGC2,ZAP70** |

**Table S6: Cell counts**

| **Cluster** | **Count** |
| --- | --- |
| CD8T_1 | 3072 |
| CD8T_3 | 2398 |
| CD8T_4 | 1853 |
| CD8T_5 | 1844 |
| CD8T_18 | 1588 |
| CD8T_6 | 1527 |
| CD8T_2 | 1463 |
| CD8T_7 | 958 |
| CD8T_8 | 540 |
| CD8T_10 | 351 |
| CD8T_12 | 298 |
| CD8T_13 | 297 |
| CD8T_15 | 187 |
| CD8T_17 | 156 |

**Table S7. Genes listed in gene heatmap**

| **TCF7**  **SELL**  **CCR7**  **LEF1**  **CD27**  **IL4R**  **IL7R**  **LTB**  **CD69**  **JUN**  **JUNB**  **CD8B**  **CD3E**  **CD3G**  **CD247**  **FOSB**  **ZAP70**  **NKG7**  **IFNG**  **PRF1**  **GNLY**  **GZMB**  **GZMA**  **GZMK**  **GZMH**  **CCL4**  **CCL5**  **KLRF1**  **KLRB1**  **KLRC1**  **KLRD1**  **KLRC3**  **KLRC4**  **FCGR3A**  **KLRG1**  **LAG3**  **HOPX**  **PRDM1**  **B3GAT1** | Transcriptional activator involved in T-cell lymphocyte differentiation.  Selectin L.  C-C Motif Chemokine Receptor 7, CD197.  Lymphoid Enhancer Binding Factor 1.  Receptor for CD70/CD27L. May play a role in survival of activated T-cells.  Interleukin 4 Receptor 4, CD124. Receptor for both interleukin 4 and interleukin 13. Couples to the JAK1/2/3-STAT6 pathway.  Interleukin 7 Receptor, CD127. Receptor for IL-7.  Lymphotoxin Beta. Cytokine that binds to LTBR/TNFRSF3. May play a specific role in immune response regulation. Provides the membrane anchor for the attachment of the heterotrimeric complex to the cell surface.  Expression of the encoded protein is induced upon activation of T lymphocytes and may play a role in proliferation.  Jun Proto-Oncogene, AP-1 Transcription Factor Subunit. Heterodimerizes with proteins of the FOS family to form an AP-1 transcription complex. Together with FOSB, plays a role in activation-induced cell death of T cells by binding to the AP-1 promoter site of FASLG/CD95L, and inducing its transcription in response to activation of the TCR/CD3 signaling pathway.  JunB Proto-Oncogene, AP-1 Transcription Factor Subunit.  CD8 Subunit Beta. In T-cells, functions primarily as a coreceptor for MHC class I molecule:peptide complex.  CD3 Epsilon Subunit Of T-Cell Receptor Complex. Part of the TCR-CD3 complex present on T-lymphocyte cell surface that plays an essential role in adaptive immune response. Plays an essential role in correct T cell development.  CD3 Gamma Subunit Of T-Cell Receptor Complex.  T-Cell Surface Glycoprotein CD3 Zeta Chain.  FosB Proto-Oncogene, AP-1 Transcription Factor Subunit. Part of the Fos gene family, FOS proteins have been implicated as regulators of cell proliferation, differentiation, and transformation.  Zeta Chain Of T Cell Receptor Associated Protein Kinase 70, When antigen presenting cells activate T-cell receptor, a series of phosphorylations lead to the recruitment of ZAP70 to the doubly phosphorylated TCR component CD247/CD3Z through ITAM motif at the plasma membrane.  Natural Killer Cell Granule Protein 7. Plays a critical role in CD8(+) T-cell and NK cell-mediated cytolysis of target cells and contributes to the cytolytic activity.  IFN-Gamma. Type II interferon produced by immune cells such as T-cells and NK cells that plays crucial roles in antimicrobial, antiviral, and antitumor responses by activating effector immune cells and enhancing antigen presentation.  Perforin 1 that promotes cytolysis and apoptosis of target cells by facilitating the uptake of cytotoxic granzymes.  Granulysin, involved in T cell activation and cytotoxicity.  Granzyme B, the encoded preproprotein is secreted by natural killer (NK) cells and cytotoxic T lymphocytes (CTLs).  Granzyme A, Abundant protease in the cytosolic granules of cytotoxic T-cells and NK-cells.  Granzyme K, this gene product is a member of a group of related serine proteases from the cytoplasmic granules of cytotoxic lymphocytes.  Granzyme H, Cytotoxic chymotrypsin-like serine protease.  C-C Motif Chemokine Ligand 4, the encoded protein is secreted and has chemokinetic and inflammatory functions.  C-C Motif Chemokine Ligand 5, Chemoattractant for blood monocytes, memory T-helper cells and eosinophils. Causes the release of histamine from basophils and activates eosinophils. May activate several chemokine receptors including CCR1, CCR3, CCR4 and CCR5.  Killer Cell Lectin Like Receptor F1. Involved in the natural killer (NK)-mediated cytolysis of PHA-induced lymphoblasts.  Killer Cell Lectin Like Receptor B1. Plays an inhibitory role on natural killer (NK) cells cytotoxicity.  Killer Cell Lectin Like Receptor C1. Key inhibitory receptor on natural killer (NK) cells that regulates their activation and effector functions.  Killer Cell Lectin Like Receptor D1.  Killer Cell Lectin Like Receptor C3.  Killer Cell Lectin Like Receptor C4.  Fc Gamma Receptor IIIa. Costimulates NK cells and trigger lysis of target cells independently of IgG binding  Killer Cell Lectin Like Receptor G1. Plays an inhibitory role on natural killer (NK) cells and T-cell functions upon binding to their non-MHC ligands.  Lymphocyte Activating 3. Following TCR engagement, LAG3 associates with CD3-TCR in the immunological synapse and directly inhibits T-cell activation.  HOP Homeobox.  PR/SET Domain 1. Transcription factor that mediates a transcriptional program in various innate and adaptive immune tissue-resident lymphocyte T cell types such as tissue-resident memory T (Trm), natural killer (trNK) and natural killer T (NKT) cells and negatively regulates gene expression of proteins that promote the egress of tissue-resident T-cell populations from non-lymphoid organs.  Beta-1,3-Glucuronyltransferase 1, CD57. Enzyme, a terminally sulfated [glycan](https://www.sciencedirect.com/topics/biochemistry-genetics-and-molecular-biology/glycan) carbohydrate epitope, identifies memory T cells that lack the capacity to proliferate. |
| --- | --- |

**Table S8. Thresholds of each antibody expression**

| **Antibody** | **Threshold** | **Antibody** | **Threshold** |
| --- | --- | --- | --- |
| CD2  CD3  CD4  CD8  CD9  CD11b  CD11c  CD14  CD16  CD19  CD20  CD24  CD25  CD27  CD33  CD36  CD38  CD43  CD45RA  CD45RO  CD56  CD69  CD86  CD95  CD123  CD126 | 2.4  2  Not used for clustering  3.7  1.3  REMOVED  2  Not used for clustering  3.25  Not used for clustering  0.25  0.6  Not used for clustering  1.15  1.3  3.4  1  2  2.15  0.8  0.5  Not used for clustering  0.9  1.85  Not used for clustering  Not used for clustering | CD127  CD137  CD141  CD142  CD152  CD154  CD163  CD183  CD184  CD185  CD192  CD194  CD195  CD196  CD197  CD206  CD223  CD273 PDL2  CD274 PDL1  CD279 PD1  CD74  IgD  IgM  SA06 TLR4  SA16 SLAN | 0.8  Not used for clustering  0.5  Not used for clustering  Not used for clustering  REMOVED  Not used for clustering  1.1  Not used for clustering  0.2  1.25  Not used for clustering  Not used for clustering  Not used for clustering  Not used for clustering  Not used for clustering  Not used for clustering  Not used for clustering  1  0.75  1.6  1.5  0.8  0.1  0.37 |

**Table S9A-L Differentially expressed genes in HighCAD vs LowCAD in all clusters.**

**A: High CAD vs low CAD DE genes in cluster 1**

| **Genes** | **avg_log2FC** | **p_val_adj** | **HighCAD_vs_LowCAD** |
| --- | --- | --- | --- |
| CCL5 | -0.5439582 | 5.11E-18 | Low_CAD |
| DUSP2 | -0.5810141 | 3.19E-11 | Low_CAD |
| IFITM3 | 0.56342791 | 2.44E-10 | High_CAD |
| LTB | 1.41457335 | 8.53E-09 | High_CAD |
| HLA.B | 0.09983714 | 3.18E-08 | High_CAD |
| CD52 | 0.1707237 | 5.04E-06 | High_CAD |
| KLRC4.KLRK1 | 0.30372196 | 4.24E-05 | High_CAD |
| GNAI2 | 0.46113718 | 5.65E-05 | High_CAD |
| TNFSF10 | 0.74343782 | 0.0001222 | High_CAD |
| KLRK1 | 0.31718553 | 0.00014009 | High_CAD |
| GZMK | -0.4014295 | 0.00049143 | Low_CAD |
| ZAP70 | 0.63266869 | 0.00168842 | High_CAD |
| CD7 | 0.5776002 | 0.00197915 | High_CAD |
| SELL | 0.299186 | 0.00306444 | High_CAD |
| TCF7 | 0.72274519 | 0.00339201 | High_CAD |
| CTSW | 0.26205734 | 0.00510221 | High_CAD |
| JUNB | 0.36591068 | 0.00510619 | High_CAD |
| NKG7 | -0.1917162 | 0.00607908 | Low_CAD |
| CST7 | -0.2669765 | 0.0424717 | Low_CAD |
| GZMA | -0.2078826 | 0.04386453 | Low_CAD |
| SELPLG | 0.21057273 | 0.04805853 | High_CAD |

**B: High CAD vs low CAD DE genes in cluster 2**

| **Genes** | **avg_log2FC** | **p_val_adj** | **HighCAD_vs_LowCAD** |
| --- | --- | --- | --- |
| CST7 | 0.3324279 | 0.00046045 | High_CAD |
| TRGC2 | 0.57400989 | 0.00086976 | High_CAD |
| NKG7 | 0.40857257 | 0.00125906 | High_CAD |
| GNAI2 | 0.61548044 | 0.00180564 | High_CAD |
| CD74 | 0.37142376 | 0.00250359 | High_CAD |
| KLRK1 | 0.30966161 | 0.00977383 | High_CAD |
| CCL4 | 0.80080115 | 0.01408158 | High_CAD |
| LTB | 0.70444724 | 0.04692155 | High_CAD |

**C: High CAD vs low CAD DE genes in cluster 3**

| **Genes** | **avg_log2FC** | **p_val_adj** | **HighCAD_vs_LowCAD** |
| --- | --- | --- | --- |
| TRGC2 | 0.71459367 | 3.64E-23 | High_CAD |
| GNLY | 0.57039718 | 4.63E-17 | High_CAD |
| IFITM3 | 0.70131687 | 9.69E-10 | High_CAD |
| GZMB | 0.61801635 | 2.17E-08 | High_CAD |
| CTSW | 0.37224802 | 5.80E-08 | High_CAD |
| TARP-refseq | 0.59318719 | 9.97E-08 | High_CAD |
| ZNF683 | 1.00602645 | 3.65E-06 | High_CAD |
| LAIR2 | 0.94980333 | 4.43E-05 | High_CAD |
| DUSP2 | -0.3490679 | 0.00047581 | Low_CAD |
| FGFBP2 | 0.32893086 | 0.0020537 | High_CAD |
| JUN | -0.6914488 | 0.00508547 | Low_CAD |
| CD160 | -0.9162007 | 0.00850055 | Low_CAD |
| HLA.B | 0.08340497 | 0.00965237 | High_CAD |
| TTC38 | 0.49327009 | 0.04212733 | High_CAD |

**D: High CAD vs low CAD DE genes in cluster 4**

| **Genes** | **avg_log2FC** | **p_val_adj** | **HighCAD_vs_LowCAD** |
| --- | --- | --- | --- |
| TCF7 | 0.96769283 | 8.28E-12 | High_CAD |
| LTB | 1.04015455 | 2.20E-07 | High_CAD |
| GNAI2 | 0.54830632 | 0.0001008 | High_CAD |
| CD7 | 0.4662666 | 0.00096477 | High_CAD |
| KLF2 | -0.3360035 | 0.00334109 | Low_CAD |
| CD8B | -0.2402863 | 0.00888539 | Low_CAD |
| JUNB | 0.41751103 | 0.00932189 | High_CAD |
| S100A9 | -0.5595662 | 0.02132994 | Low_CAD |

**Table SE: High CAD vs low CAD DE genes in cluster 5**

| **Genes** | **avg_log2FC** | **p_val_adj** | **HighCAD_vs_LowCAD** |
| --- | --- | --- | --- |
| TRGC2 | 0.75182735 | 6.81E-17 | High_CAD |
| GNLY | 0.38985204 | 8.07E-15 | High_CAD |
| HLA.B | 0.17504853 | 5.07E-14 | High_CAD |
| CTSW | 0.53918642 | 2.73E-13 | High_CAD |
| GNAI2 | 0.77058919 | 2.02E-08 | High_CAD |
| GZMK | -0.6994889 | 4.21E-08 | Low_CAD |
| CCL5 | -0.2632845 | 9.99E-08 | Low_CAD |
| CD8B | 0.4603099 | 1.22E-06 | High_CAD |
| GZMH | 0.31024903 | 4.70E-06 | High_CAD |
| IL32 | -0.2221478 | 5.34E-06 | Low_CAD |
| ZNF683 | 0.92754199 | 6.34E-06 | High_CAD |
| RGS1 | -1.1030177 | 1.17E-05 | Low_CAD |
| DUSP1 | -0.5094624 | 1.27E-05 | Low_CAD |
| FGFBP2 | 0.36448796 | 6.47E-05 | High_CAD |
| ZAP70 | 0.79638128 | 8.39E-05 | High_CAD |
| B3GAT1 | 0.94776104 | 0.000256 | High_CAD |
| KLRK1 | 0.40740621 | 0.00027849 | High_CAD |
| TARP-refseq | 0.50266383 | 0.00038754 | High_CAD |
| S100A9 | -0.7674793 | 0.00093975 | Low_CAD |
| HLA.DRA | -0.7772877 | 0.00121935 | Low_CAD |
| CD48 | -0.30988 | 0.00368133 | Low_CAD |
| GZMB | 0.25670951 | 0.0067004 | High_CAD |
| IFITM3 | 0.41390943 | 0.00677268 | High_CAD |
| FOSB | -0.6961639 | 0.008266 | Low_CAD |
| PXN | 0.28881685 | 0.00895321 | High_CAD |
| TTC38 | 0.66360477 | 0.01324316 | High_CAD |
| CD7 | 0.69330754 | 0.03070109 | High_CAD |

**E: High CAD vs low CAD DE genes in cluster 6**

| **Genes** | **avg_log2FC** | **p_val_adj** | **HighCAD_vs_LowCAD** |
| --- | --- | --- | --- |
| TRGC2 | 0.45194065 | 1.28E-06 | High_CAD |
| ZAP70 | 0.74182359 | 2.43E-06 | High_CAD |
| HLA.B | 0.10137268 | 0.00091298 | High_CAD |
| GNAI2 | 0.49642712 | 0.00196683 | High_CAD |
| IL32 | 0.1698638 | 0.00216042 | High_CAD |
| TARP-refseq | 0.29915105 | 0.00220067 | High_CAD |
| CTSW | 0.27806363 | 0.00222965 | High_CAD |
| GNLY | -0.2139023 | 0.02947189 | Low_CAD |
| CTSA | 0.41333521 | 0.04886917 | High_CAD |

**G: High CAD vs low CAD DE genes in cluster 7**

| **Genes** | **avg_log2FC** | **p_val_adj** | **HighCAD_vs_LowCAD** |
| --- | --- | --- | --- |
| HLA.B | 0.1466726 | 0.00104868 | High_CAD |
| GZMH | -0.5631823 | 0.01472399 | Low_CAD |
| TRGC2 | 0.6534631 | 0.01869049 | High_CAD |
| CD160 | -1.2985546 | 0.04376843 | Low_CAD |

**H: High CAD vs low CAD DE genes in cluster 8**

| **Genes** | **avg_log2FC** | **p_val_adj** | **HighCAD_vs_LowCAD** |
| --- | --- | --- | --- |
| GNAI2 | 1.83029208 | 1.04E-09 | High_CAD |
| LAIR2 | 2.11772703 | 5.67E-07 | High_CAD |
| CTSW | 0.56430546 | 0.00010189 | High_CAD |
| HLA.B | 0.18240663 | 0.00010678 | High_CAD |
| TRGC2 | 0.46838881 | 0.00012154 | High_CAD |
| IFITM3 | 0.91549403 | 0.01006006 | High_CAD |
| B3GAT1 | 1.2664273 | 0.03524902 | High_CAD |

**I: High CAD vs low CAD DE genes in cluster 10**

| **Genes** | **avg_log2FC** | **p_val_adj** | **HighCAD_vs_LowCAD** |
| --- | --- | --- | --- |
| TRGC2 | 0.82848481 | 0.00966663 | High_CAD |
| LAIR2 | 2.57297904 | 0.01154911 | High_CAD |

**J: High CAD vs low CAD DE genes in cluster 12**

| **Genes** | **avg_log2FC** | **p_val_adj** | **HighCAD_vs_LowCAD** |
| --- | --- | --- | --- |
| IL32 | 0.50942452 | 7.34E-06 | High_CAD |
| TRGC2 | 0.65156816 | 0.03568887 | High_CAD |

**K: High CAD vs low CAD DE genes in cluster 13**

| **Genes** | **avg_log2FC** | **p_val_adj** | **HighCAD_vs_LowCAD** |
| --- | --- | --- | --- |
| CD8B | -1.2455125 | 0.01487734 | Low_CAD |

**L: High CAD vs low CAD DE genes in cluster 18**

| **Genes** | **avg_log2FC** | **p_val_adj** | **HighCAD_vs_LowCAD** |
| --- | --- | --- | --- |
| CCL5 | -0.2819669 | 9.62E-06 | Low_CAD |
| KLRB1 | -0.9624165 | 1.71E-05 | Low_CAD |
| CD8B | 0.33123776 | 0.00017342 | High_CAD |
| TCF7 | 0.86868221 | 0.00027156 | High_CAD |
| GNAI2 | 0.76053114 | 0.0004167 | High_CAD |
| LTB | 1.31683511 | 0.0114303 | High_CAD |
| KLRK1 | 0.29701777 | 0.01800414 | High_CAD |
